# Role of Environmental Factors in Legume-*Rhizobium* Symbiosis: A Review

**DOI:** 10.3390/biom15010118

**Published:** 2025-01-14

**Authors:** Liudmyla Yeremko, Katarzyna Czopek, Mariola Staniak, Mykola Marenych, Volodymyr Hanhur

**Affiliations:** 1 Department of Crop Production, Poltava State Agrarian University, Skovoroda St., 1/3, 36000 Poltava, Ukraine; liudmyla.yeremko@pdau.edu.ua (L.Y.); volodymyr.hanhur@pdau.edu.ua (V.H.); 2 Department of Crops and Yield Quality, Institute of Soil Science and Plant Cultivation—State Research Institute, 8 Czartoryskich St., 24-100 Pulawy, Poland; staniakm@iung.pulawy.pl; 3 Department of Breeding, Seed Production and Genetics, Poltava State Agrarian University, Skovoroda St., 1/3, 36000 Poltava, Ukraine; mykola.marenych@pdau.edu.ua

**Keywords:** legumes, rhizobia, biological nitrogen fixation (BNF), abiotic stress, soil nutrients, sustainable agriculture, protein crops

## Abstract

Legumes play a pivotal role in addressing global challenges of food and nutrition security by offering a sustainable source of protein and bioactive compounds. The capacity of legumes to establish symbiotic relationships with rhizobia bacteria enables biological nitrogen fixation (BNF), reducing the dependence on chemical fertilizers while enhancing soil health. However, the efficiency of this symbiosis is significantly influenced by environmental factors, such as soil acidity, salinity, temperature, moisture content, light intensity, and nutrient availability. These factors affect key processes, including rhizobia survival, nodule formation, and nitrogenase activity, ultimately determining the growth and productivity of legumes. This review summarizes current knowledge on legume-rhizobia interactions under varying abiotic conditions. It highlights the impact of salinity and acidity in limiting nodule development, soil temperature in regulating microbial community dynamics, and moisture availability in modulating metabolic and hormonal responses during drought and waterlogging. Moreover, the role of essential nutrients, including nitrogen, phosphorus, potassium, and trace elements such as iron, molybdenum, and boron, in optimizing symbiosis is critically analyzed.

## 1. Introduction

In solving global food and nutrition security problems, the cultivation of legumes as the main source of highly nutritious protein resources is gaining strategic importance. It is well known that protein, as an important nutrient, plays a key role in the physiological and biochemical reactions in the organism necessary to ensure the body’s growth and development [1,2]. In the structure of protein resources used for nutrition, proteins of animal origin play a significant role. However, according to the results of scientific research, the presence of a large amount of meat in the human diet can lead to obesity and disorders of the functioning of the cardiovascular system related to it. On the other hand, the so-called hidden hunger caused by insufficient micronutrients in the daily diet leads to many health problems, especially for children. They can be manifested in stunted growth, poor weight gain, cognitive impairment, and mental disorders [3,4]. One of the components that can ensure a healthy diet for the population of both developed and developing countries can be highly nutritious plant products made from legume seeds [5].

The main constituent components of the legume seeds are protein, complex carbohydrates, dietary fiber, vitamins, minerals, and biologically active compounds. Legume seeds are characterized by the absence of cholesterol and, with the exception of soybeans, chickpeas, white lupine, and peanuts, contain a low amount of lipids [6]. Biologically active compounds of legumes (including phytoestrogens, saponins, phenolic compounds, oligosaccharides, and alkaloids) are characterized by antithrombotic, antioxidant, opioid-like activity, have cytomodulatory or immunomodulatory effects, and contribute to the improvement of mineral bioavailability [7,8,9]. Pharmaceuticals based on their raw materials are widely used in the prevention of occurrence and medical therapy of cardiovascular, cancer diseases, diabetes, and obesity [10,11]. At the same time, legumes are a valuable source of highly nutritious feed resources used in livestock farming [12,13].

Cultivation of legumes improves the biological, physical and chemical characteristics of the soil [14,15], contributes to the reduction in greenhouse gasses in the atmosphere [16], and limits the occurrence of diseases, pests, diseases, and nematodes by disrupting the biological cycle of their development [17]. Legumes have a unique biological property, which consists of establishing symbiotic relationships with nitrogen fixing bacteria of the genera *Rhizobium*. Kebebe [18] notes that in the process of legume–rhizobia symbiosis, legumes can fix about 100 to 300 kg ha^−1^ of atmospheric nitrogen (N_2_) annually, thus providing about 139–175 million tons of nitrogen to the soil. This, in turn, reduces the cost of applying about 80–90 tons of mineral nitrogen fertilizers annually [19]. Nitrogen (N) is an element that occurs mainly in the atmosphere, lithosphere, and biosphere in various degrees of oxidation [20,21]. Only about 2% of this element is found in living organisms and their biocenoses, with the remainder occurring in inorganic form [22]. Nitrogen in the gaseous form (it makes up about 78% of the atmospheric composition) is very persistent due to the triple covalent bond between N atoms, but in this form, it is unabsorbable by most higher organisms and unreactive [23]. In the bioavailable form, N is found in soil in the form of organic and inorganic compounds such as nitrates –NO_3_, nitrites –NO_2_, and ammonium ions –NH_4_ [24,25]. Biological nitrogen fixation (BNF) is one of the most important processes on Earth. It involves the conversion of N_2_, which is not assimilated by plants, into ammonia, the form available to plants [26,27]. This process is a highly energetic transformation, requiring reducing forces, i.e., electrons and protons (H^+^), as well as large amounts of energy (ATP) [28]. The symbiotic relationship between rhizobia and legumes plays a key role in sustainable agriculture by facilitating access to N_2_, improving soil fertility and reducing the need for chemical fertilizers.

**Figure 1:**
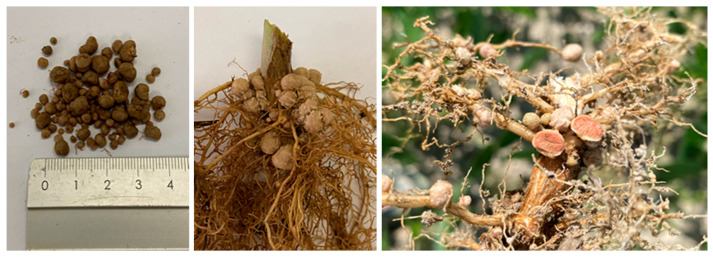
Nodules on soybean roots [Photo: K. Czopek].

After the mutualistic relationship between the two symbiosis partners is established, the roots of plants form structures known as root nodules, in which MgATP-dependent N_2_ reduction by the nitrogenase complex occurs (Figure 1).

The produced ammonia is used by plants as a source of N in the synthesis of organic compounds. To support the bacterial endosymbionts’ metabolism, photosynthetic products are supplied from the plant to nodules [29]. The establishment of the legume–rhizobia symbiosis as an N_2_-fixing system is interconnected with the physiological status of the plant host and is determined by the influence of environmental factors, such as soil type, temperature, moisture, pH, salinity, micro, and macronutrient content [19,30,31]. These factors can affect various aspects of symbiosis, including rhizobia survival in the soil, infection process, legume development, nodule function, and, indirectly, the growth of the host plant [32]. Due to symbiosis, legumes cover their nutritional needs in relation to N and, therefore, have lower requirements for mineral fertilization, which is limited to the necessary minimum, generally a small pre-sowing (starter) dose. This allows deficiencies of the nutrient to be made up in the initial period of plant growth and development, when the BNF process has not yet begun [33]. Individual legume species can only coexist with a specific bacterial species, for example, beans (*Phaseolus vulgaris* L.) have the ability to symbiosis with *Rhizobium leguminosarum* bv. *phaseoli*, and soybean (*Glycine max* (L.) Merr.) with *Bradyrhizobium japonicum* (Table 1). Bacteria coexisting with legumes do not always occur naturally in the soils in which these species are grown, so it is sometimes necessary to inoculate legume seeds prior to sowing with appropriately selected bacterial strains to enable the rapid colonization of the rhizosphere and ensure effective nodulation, thereby improving the BNF process and maximizing yield [34,35,36]. However, the majority of soils used in agriculture contain rhizobia populations that compete in the plant host nodulation process with strains introduced by inoculation.

The aim of this review is to summarize the current state of knowledge regarding the symbiosis between legumes and rhizobia bacteria and to identify key environmental factors affecting the efficiency of biological nitrogen fixation (BNF). Particular focus is placed on the impact of factors such as soil salinity, acidity, temperature, moisture, light intensity, and the availability of macro- and micronutrients. This review seeks to highlight the challenges and opportunities associated with optimizing the symbiosis, which can enhance legume productivity and support sustainable agricultural practices.

## 2. Soil Salinity

One of the factors that determine the physiological status of plants and the formation and functioning of legumes with rhizobia symbiosis is the presence of salts in the soil. The negative effect of soil salinity on plant growth and development manifests in a decrease in the intensity of growth processes and BNF due to a decrease in photosynthetic activity [19]. At the same time, soil salinity can lead to a decrease in the population of soil microorganisms, distortion of their structure, as well as cause inhibition of root and root hair development, and disappearance of the mucous layer on them. This, in turn, leads to a deterioration of bacterial survival and a decrease in the possibility of forming an infectious thread during nodulation, a decrease in the intensity of nodule respiration, and a decrease in the production of leghemoglobin [32].

In the course of scientific research in the variants of inoculation of soybean seeds with *B. japonicum* in the presence of 170 mM NaCl, a slight twisting or deformation of root hairs was observed, while in the case of increasing the NaCl concentration to 210 mM, nodules on soybean roots were not formed. At the same time, studies have shown different effects of salt on the development and vital activity of bacteria of the *Rhizobium* and *Bradyrhizobium* strains. Thus, in the presence of 100 mM NaCl, a slowdown in the growth of *B. japonicum* was observed, while *R. meliloti* proved to be quite resistant to the effects of increased to 300–700 mM NaCl concentration [19].

The rhizobia osmotolerance is mainly related to the presence of accumulated or unaccumulated osmoprotectants (betaine, amino acids, and sugars) [38,39]. They are low-molecular-weight organic solutions and are characterized by the effect of counteracting dehydration caused by low water activity in the environment [40]. Some authors noted that in some cases, the interaction of plant roots with bacteria characterized by high salt tolerance (which is manifested in the ability to grow in the range of 300–700 mM NaCl) leads to the formation of inefficient nodules with a low rate of nitrogen fixation [32].

## 3. Soil pH

A factor that significantly limits the cultivation of legumes in the world is soil acidity. In general, soils with an indifferent or weakly alkaline pH are favorable for legumes, while an acidic environment prevents most legume species from initiating the symbiosis process. Low soil pH is caused by an excess of acidic cations, such as H^+^, Al^3+^, and Mn^2+^ compared to alkaline cations, such as Ca^2+^, Mg^2+^, K^+^, and Na^+^. Its negative impact is reflected in the damage to the root system of plants and the corresponding weakening of the absorption and assimilation of water and nutrients dissolved in it, resulting in yield losses in legumes of up to 50% [41].

In acidic soils, the release of carbon compounds by the plant root system, which are substrates for soil microorganisms living in the rhizosphere, is significantly reduced. Due to a significant limitation of rhizobia survival, as well as a negative impact on nodule formation in acidic soils, BNF is significantly reduced [42]. The negative effect of soil acidity is manifested already in the early stages of the infection process, namely during the exchange of signals between the host plant and the microsymbiont, which negatively affects the attachment of rhizobia to root hairs and, accordingly, leads to a decrease in bacterial colonization of roots [19]. Ferguson et al. [41] attributed this effect to a decrease in flavonoid secretion into the rhizosphere by the root system of legumes, which leads to a decrease in the induction of the Nod gene by rhizobia, inhibition of nod factor secretion and excretion of Nod metabolite. In turn, the decrease in nod factor signaling leads to deformation of root hairs and their twisting. At the same time, cell division and nodule primordial tissue formation are inhibited [43].

## 4. Soil Temperature

An important environmental factor that determines the diversity of bacterial communities, as well as the formation of plant-bacteria relationships, is soil temperature [44]. Numerous studies have shown that soil temperatures that are higher or lower than the optimum, have a negative impact on the relationship between both partners of the symbiosis, in general. The optimal range of soil temperature for the functioning of nodule bacteria is between 28 and 31 °C [19]. However, some strains are able to survive an increase in soil temperature up to 35–40 °C [45]. The range of temperature optimum for legume–rhizobia symbiosis is determined by the biological characteristics of both partners [46]. The results of Michiels et al. [47] showed that the optimal soil temperature for BNF in soybean and peanut is 30 °C, and the critical temperature is in the range of 35–40 °C. The most favorable temperature regime for the root zone for BNF of common beans ranges from 25 to 30 °C. Increasing the soil temperature to 30–33 °C inhibits legume–rhizobia symbiosis, which is manifested in a smaller size and weight of nodules, as well as in a decrease in nitrogenase activity [19]. Ramires and Damo [48] reported that an increase in temperature in the root zone is critical for BNF due to the deterioration of the physiological state of rhizobium, reducing their ability to infect root hairs, form infectious threads, initiate nodule formation and growth, reduce the content of leghemoglobin in nodules, and reduce the activity of the nitrogenase complex.

On the other hand, low temperatures may limit the survival of rhizobia, as well as the secretion of flavonoids by legumes, hindering the exchange of specific signaling molecules between them, which ultimately leads to a reduction in the number of nodules and adversely affects legume growth [49]. According to various studies, the effect of cold stress is manifested in damage to bacterial cell membranes [50] and stabilization of secondary RNA/DNA structures, which in turn leads to a decrease in the efficiency of the main stages of the implementation of genetic information (translation, transcription, and replication) [51]. At the same time, signalization between both partners of the legume-rhizobia symbiosis is disrupted, the period of nodule initiation and formation is prolonged [52], and their size is reduced [53].

## 5. Soil Moisture Content

The moisture availability of plants is a key environmental factor that determines the conditions of vital activity not only in microbial communities, but also in plants. It plays a key role in biological processes occurring in plants at all stages of development [54]. Álvarez-Aragóon et al. [55] report that the aboveground and underground parts of plants respond to drought conditions in different ways. Thus, if the aboveground part undergoes significant modifications aimed at reducing moisture loss and optimizing metabolism, changes in the root system contribute to better moisture supply to plants [56]. Dehydration of plant cells and tissues caused by water deficit leads to a decrease in photosynthetic activity, efficiency of moisture used by plants, absorption, and transport of nutrients from roots to shoots, inhibition of synthesis of organic compounds, as well as changes in the hormonal balance of plants [57]. The morphological manifestation of these changes is a decrease in root proliferation, leaf blade size, and internode length, which consequently leads to a decrease in plant productivity [58].

Drought affects the initiation of symbiosis because water deficit inhibits the ex-change of signaling molecules involved in communication between the legume and rhizobia, resulting in poor nodulation and reduced BNF [59,60,61]. Furlan et al. [62] note that insufficient moisture supply of plants negatively affects the density of populations of nitrogen-fixing microorganisms and leads to disruption of the process of infection of root hairs due to inhibition of the formation of infectious threads [63,64]. The main physiological responses to water stress in legume–rhizobia systems are a reduction in the intensity of carbon metabolism in nodules, reduced nitrogenase activity due to oxygen limitation, regulation of feedback by accumulation of nitrogen fixation products, and modification of rhizobia cells [19,65,66,67,68].

Numerous studies confirm the reduction in legume nodulation under drought stress [69,70]. Michałek [71] showed a reduction in the number, weight, and activity of nodules formed on the roots of soybean plants at the flowering stage under the influence of periodic drought, which had a significant impact on plant productivity. Soil water content of 50% and 25% FWC reduced, compared to the control, the number of nodules by 22 and 42%, root fresh weight by 46 and 69%, and root dry weight by 23 and 41%, respectively. Also, Sadeghipour and Abbasi [72] showed a decrease in symbiotic bacteria activity and a decrease in the number of pods per plant, seeds per pod, seed weight, and seed yield under drought stress conditions.

The nodulation process is adversely affected by both deficiency and excess water. Increased soil moisture stimulates anaerobic processes that lead to the appearance of phytotoxins, compounds produced by anaerobic microorganisms in the soil. These chemical compounds can negatively affect plant roots, restricting their growth, decreasing nutrient transpiration, damaging roots, and impairing the ability to form root nodules. These compounds are also not indifferent to rhizobia populations [73]. Particularly sensitive to fluctuations in soil moisture and oxygen content are nitrogenases, whose synthesis is inhibited in an environment with high oxygen content. Higher soil moisture creates a low-oxygen environment for N_2_-fixing bacteria, which may favor BNF productivity [74].

## 6. Light

Light is the main source of energy that drives the process of photosynthesis, during which plants convert light energy into energy from chemical bonds contained in sugar and other organic compounds [75].

Organic compounds synthesized during photosynthesis act as a primary source of carbon for plants and their excess is released into the rhizosphere in the form of root exudates [76]. Sugars, organic acids, amino acids, and secondary metabolites released into the rhizosphere play an important role in providing microorganisms with carbon and energy, thus determining the number of rhizosphere communities and establishing mutualistic relationships between legume plants and microorganisms [77]. At the same time, photosynthetic products act as a source of energy for legume–rhizobia symbiosis. According to Lepetit and Brouquisse [29], more than 25% of photosynthetic products are used by plants to support the functioning of BNF.

## 7. Nutrient Content in the Soil

An important factor determining the efficiency of legume–rhizobia symbiosis is the presence of mineral nutrients in the soil [44].

**Nitrogen (N)** is one of the essential macronutrients that determines the growth and development of the vegetative part of plants. This element is required for numerous processes of synthesis of organic compounds, including proteins [78], chlorophyll, nucleic acids, and hormones [42,79]. Plants can absorb it from mineralized soil organic matter [80], industrial nitrogen fertilizers [81], and the BNF process. Abd-Alla et al. [82] note that if there is a sufficient amount of inorganic (mineral) nitrogen (NO_3_^−^) in the soil, plants absorb nitrate from the soil as non-symbiotic higher plants. Scientists attribute this effect to a reduction in energy consumption by plants for the N_2_ to NH_3_ conversion by the nitrogenase complex in nodules. It is known that for its transformation, the plant uses organic compounds synthesized during photosynthesis. Their amount is equivalent to 25% of shoot dry matter at harvest. Therefore, in order to reduce these costs, if other alternative sources of N are available, legumes will use them to ensure their vital activity. Under conditions of mineral N deficiency in the soil, plants maintain BNF in nodules at the lowest level necessary to ensure growth processes [82].

Mineral N has an inhibitory effect on the interaction of legume–rhizobia symbiosis partners [83]. This is indicated by a decrease in the colonization of root hairs by N-fixing microorganisms [66], weakening of root hair twisting [84], reduction in the number of infectious threads, and the associated inhibition of nodule development and disruption of the BNF process [85,86]. In addition, the presence of mineral N causes early nodule senescence and reverse inhibition of nitrogenase activity [87]. In legumes, the mineral N content of the soil is particularly important at the beginning of vegetation before root nodules appear and bacterial symbiosis develops. From the budding stage onward, when the assimilation of N_2_ from the air by rhizobia begins, legumes are self-sufficient in this nutrient. Therefore, N deficiency may be related to disruptions in the establishment of nodules, their function, or N transport to vegetative parts. A study by Salvagiotti et al. [80] showed that about 80 kg N ha^−1^ is needed to produce 1 t ha^−1^ of soybean seed. This requirement is met 50–80% by the plant through the BNF pathway [88]. Therefore, if higher yields with satisfactory seed quality are desired, soil N and BNF may not be sufficient to meet the needs of the plant during the seed-filling period to realize the yield potential of soybean.

**Phosphorus (P)** plays a key role in the energy supply of physiological processes of plant life, in particular, the BNF process [89]. This element is essential for nodule growth and proper symbiosis with rhizobia bacteria. Root nodules typically contain two to three times more P than other plant organs [90,91]. When plants are sufficiently supplied with P, photosynthetic products are used to support both root development and nodule function [92,93]. Under P deficiency, on the contrary, most of the synthesized organic compounds are directed to the roots rather than to the nodules. An insufficient supply of P to plants leads to the inhibition of the nodulation process [94,95], nitrogenase activity, a reduction in nodule leghemoglobin content [96], a decrease in active N_2_-fixation in nodules [26], and the relevant deterioration of BNF in numerous studies confirm that P influences earlier formations of active root nodules, increases their size and number, and improves the amount of bioavailable N per unit weight [97,98], in addition to increasing root and nodules weight [99,100].

**Potassium (K)** is an important element that determines the adaptive capacity of plants to abiotic stresses. K acts as an activator of more than 60 enzyme systems that catalyze numerous metabolic reactions in plants. It maintains the osmotic status and turgor of cells, regulates their cation-anion balance and cytoplasmic pH, controls membrane polarization, cell expansion, and stomatal movements, thus regulating the supply of CO_2_ and moisture to the plant [101]. In legumes, K, as the most abundant intracellular cation, plays an important role in the formation of a powerful root system and its moisture absorption, root hair growth, which in turn improves nodulation and BNF [44]. At the same time, its presence provides support for the turgor pressure of bacterial cells, pH regulation, gene expression, and activation of cellular enzymes [102].

**Sulfur (S)** is an element that has an important function in nitrogen management and is involved in the biosynthesis and function of molybdenum-containing enzyme structures (nitrogenase enzyme complex). As a component of the amino acids Cys and Met, metal cofactors, coenzymes, and secondary metabolites, take part in the processes of synthesizing proteins and chlorophyll, nutrient absorption, and nitrogen use efficiency by plants during their growth and development, as well as plant resistance to various stress factors [103]. Its deficiency negatively affects the formation of legume–rhizobia symbiosis, which is associated with inhibition of nodulation and BNF, due to a decrease in the intensity of biosynthesis and the activity of nitrogenase, that contains an increased amount of S in the form of FeS clusters [104]. Some authors attribute this to a decrease in the availability of Cys and Met amino acids and a general change in nodule metabolism. The reduction in S supply to plants leads to a limited supply of energy and carbon skeleton to the legume–rhizobia symbiosis, due to a decrease in the content of leghemoglobin and glucose in nodules, ATP in mitochondria and bacteroids, and ferredoxin in bacteroids [105,106,107]. Ferredoxin and nitrogenase are crucial for BNF process, as they contain the iron-sulfur and molybdenum-iron-sulfur metal clusters [108]. S can affect BNF by modulating the growth and function of root nodules or by influencing the growth of the host plant. S deficiency reduces BNF efficiency in legumes by decreasing ferredoxin and leghemoglobin concentrations and ATP supply [109].

**Calcium (Ca)** influences the proper development of the root system and the aboveground parts of the plant. Through adequate root growth, it increases the likelihood of nodules forming and their number on the roots. In the symbiotic association between legumes and rhizobia, Ca plays a crucial role in signaling between plants and bacteria and recognition of rhizobial nodulation factors in the early stages of symbiotic interaction [44]. Miwa et al. [110] associate this with an increase in the level of flavonoids in plant root exudates, which in turn enhances the induction of nodal genes through signal perception by rhizobia. Ca enhances the colonization of the legume root system by rhizobia due to an increased release of bacterial exopolysaccharides and their gel formation as a means of attaching rhizobia to root hairs [42]. At the same time, when plants are sufficiently supplied with this element, an increase in the activity of Nod genes is observed [111].

**Magnesium (Mg)** plays a significant role in the growth processes of both the legumes and the rhizobia. At the beginning of the vegetation period, this element is essential for proper growth of the root system, which later determines adequate water and nutrient uptake. In the plant, the importance of Mg for energy metabolism, photosynthesis, protein synthesis, carbon allocation, and stress resistance through the Mg transporter is well known [112]. Mg also has a beneficial effect on the metabolism and distribution of N in legumes, which translates into planning levels and seed protein content. Mg affects nodule formation by regulating C-N transport in nodules and by changing the plasmodermal permeability of internal cortical cells in nodules [112,113,114].

**Iron (Fe)** is a key component of the nitrogenase complex, which is a MoFe protein, as well as a cofactor of other proteins that are important for N_2_ fixation, such as leghemoglobin, cytochrome, or ferredoxin [115]. In plants, Fe deficiency leads to a decrease in chlorophyll synthesis and inhibition of photosynthesis, as well as causing chlorosis and serious growth disorders in plants [116]. Excess iron is harmful and can negatively affect the uptake of manganese by plants.

**Molybdenum (Mo)**, which is a component of enzymes, plays an important role in key metabolic processes of plants, in particular, photosynthesis and protein synthesis [117]. Mo, as a constituent part of the FeMoCo nitrogenase complex, plays a key role in the process of nitrogen reduction [118]. Therefore, the amount of this element in the nodules is about 10 times higher than in other parts of the plant [119]. Mo stimulates the formation of new root nodules and N_2_ fixation and influences the number of flowers and pods on the plant, which has a direct effect on seed yield. Deficiency of the Mo in the soil leads to disorganization of biochemical and physiological reactions, which weakens the growth of plants, reduces the seed yield, and deteriorates its quality indicators [120]. A decrease in molecular nitrogen fixation caused by Mo deficiency leads to N deficiency in legumes [121]. Mo is also involved in the metabolism of P, S, and Fe. A water deficit results in a reduction in BNF and, when Mo content in the soil is low, it can reduce the amount of N transformed up to several kg ha^−1^ [122].

**Boron (B)** is an essential component of cell walls, thus increasing stem resistance to cracking and secondary pathogen infestation. B is involved in reproduction, phenolic metabolism, and N_2_ fixation. B plays a key role at every stage of nodule development and morphogenesis. Redondo-Nieto et al. [123] point out that it is important for maintaining the structure of rhizobia cell membranes and for the exchange of signals between plants and bacteria. At the same time, its role in the processes of rhizobia infection of legume roots, nodule invasion, and bacteroid development is well known. Deficiency of B reduces the formation and growth of root nodules, resulting in N deficiency, weaker plant growth, and reduced plant flowering time.

**Nickel (Ni)** is an activator of the urease enzyme, which facilitates the hydrolysis of urea to produce NH_3_ [124]. As an integral part of the hydrogenase, nickel takes part in the process of deactivation of hydrogen gas, which is a by-product of BNF. The utilization of gaseous hydrogen increases the energy efficiency of N_2_ fixation by producing more ATP and, simultaneously, reducing energy use. At the same time, this process mitigates the inhibitory effect of gaseous hydrogen and oxygen on nitrogenase activity [125].

**Cobalt (Co)** plays an important role in the development, morphological changes, and vital functions of rhizobia, as well as in the differentiation of bacteroids [125]. This element is a constituent of cobalamin, which is a cofactor of rhizobia enzymes that act as catalysts for the conversion of N_2_ to NH_3_^−^ [126]. The research results indicate a significant role of Co fertilization in increasing the efficiency of legume–rhizobia symbiosis, as well as in increasing the level of N content in legume tissues [125]. At the same time, some authors report a decrease in the concentration of reactive oxygen species in root nodules, which improves the growth conditions of rhizobia [127].

**Zinc (Zn)** is an element involved in carrying out efficient and effective photosynthesis, and it is involved in the reduction in free radicals, which are formed during the action of various stress factors. It acts as a catalytic cofactor and a structural element in a large number of proteins [128]. Its deficiency leads to significant changes in metabolism, which is expressed in the inhibition of growth processes and defects in the formation of the aboveground part of plants, in particular, chlorosis between the fibers, the appearance of necrotic areas on the leaves, reduction in internode length and leaf size, bending of the leaf blades [129], and reduction of nitrogenase activity [130]. Zn also has a yield-forming effect by stimulating the biosynthesis of plant hormones, including auxins, whose deficiency causes plant growth inhibition. Weisany et al. [131] noted that Zn fertilization of plants growing on saline soils plants increases the intensity of photosynthesis and the efficiency of soil moisture utilization by plants.

**Manganese (Mn)** plays a crucial role in a variety of physiological and biochemical processes in plant life. It is an integral part of more than 35 key enzymes in photosynthesis, respiration, nitrogen metabolism, and the processes of proteins, lipids, carbohydrates, and chlorophyll synthesis [125,132]. In many enzymes, Mn is interchangeable with Ca, Co, Cu, Mg, or Zn. Its importance in the process of assimilation of other mineral nutrients (Fe, Ca, and Mg) by plants is widely known. In addition, it is involved in the conversion of nitrate (NO_3_^−^) to ammonia (NH_3_) [125]. As a cofactor of the antioxidant enzyme superoxide dismutase, it has a protective effect on root nodules under the influence of oxidative stress caused by reactive oxygen species [133]. To ensure normal functioning, plants need only a small amount of this trace element, but its deficiency can lead to delayed root growth and reduced nutrient uptake, disruption of hormonal signaling, reduced immune response to adverse abiotic factors, and pathogen damage [134].

**Copper (Cu),** as a cofactor of enzymes, plays a key role in photosynthesis, respiration, supply, and the distribution of photoassimilates between plant organs, ethylene signaling, regulation of free radical levels [135,136], and energy transfer to bacteroids [125]. Cu is a component of enzymes associated with free radical metabolism, respiration, rhizobia, and vital functions in root nodules [125].

Numerous studies show that Cu and Mn reduce the activity of rhizobia, interfere with the formation of new nodules, and inhibit bacterial activity in already-formed nodules [137,138,139]. Cu has also been shown to have bactericidal effects [140]. These results relate to the detrimental effect of Cu and Mn on rhizobia bacteria with which legume seeds have been treated in the form of sulfate or chloride [137,138,139,140,141]. These micronutrients already inhibit bacterial activity when applied to inoculated seeds [36]. The effect of these micronutrients in the chelated form on the activity of symbiotic bacteria has not yet been investigated.

The comparison of the influence of various nutritional components on the biological nitrogen fixation process has been presented in a summary table (Table 2). Based on the number of publications on each component, it can be inferred that some components have been thoroughly studied, while there is limited literature available for others. Additionally, the effects of certain components, particularly copper and manganese, vary significantly, highlighting the need for further research on the impact of these components on the BNF process.

## 8. Conclusions

The review highlights the multifaceted role of legumes in sustainable agriculture and their ability to mitigate environmental challenges while providing high-quality protein sources. The symbiosis between legumes and rhizobia bacteria is crucial for biological nitrogen fixation (BNF), contributing significantly to soil fertility and reducing the dependency on synthetic nitrogen fertilizers. However, the efficiency of this process is highly influenced by environmental factors, including soil salinity, acidity, temperature, moisture, light availability, and nutrient content. Addressing these challenges requires a comprehensive understanding of the mechanisms underlying legume–rhizobia interactions and their response to abiotic stresses. Innovations in seed inoculation, soil management practices, and nutrient supplementation can improve BNF efficiency and crop yields. The economic and environmental benefits of legumes underscore their importance in sustainable agricultural systems, where they enhance soil health, support biodiversity, and reduce greenhouse gas emissions.

Future research should focus on breeding stress-tolerant legume varieties and developing advanced inoculants to maximize BNF under diverse environmental conditions. This holistic approach will enable the full utilization of the potential of legumes, contributing to global food security and environmental sustainability.

## Figures and Tables

**Table 1:** Examples of symbiotic interactions between bacteria rhizobia and legume [37].

Genera	Species	Host-Legume
*Rhizobium*	*R. leguminosarum* bv. *viciae* *R. leguminosarum* bv. *phaseoli* *R. leguminosarum* bv. *trifolii* *R. leguminosarum* bv. *etli*	vetch, peas, lentils, chickling vetch beans clover beans, common bean
*Bradyrhizobium*	*B. japonicum* *B. elkani*	soybean common bean
*Sinorhizobium*	*S. meliloti* *S. fredii*	melilot, alfalfa, fenugreek soybean, common bean
*Mezorhizobium*	*M. loti* *M. huakuii*	lotus, lupin Chinese milk vetch

**Table 2:** The influence of nutrients in the soil on the BNF process.

Element	Effect	References
**Nitrogen**	synthesis of organic compounds	[78]
	synthesis of chlorophyll, nucleic acids, and hormones	[42,79]
	decrease in the colonization of root hairs by N-fixing microorganisms	[66]
	early nodule senescence and inhibition of nitrogenase activity	[87]
	weakening of root hairs twisting	[84]
	reduction in the number of infectious threads and associated inhibition of nodule development and disruption of the BNF process	[83,85,86]
**Phosphorus**	providing energy for physiological processes	[89]
	root nodule growth and proper symbiosis with rhizobia	[90,91,94,95,96]
	growth and development of roots and root nodules	[92,93,97,98,99,100]
**Potassium**	supply of CO_2_ and moisture to the plant	[101]
	growth and development of roots and root hairs	[44]
	activation of cellular enzymes; maintaining the appropriate turgor pressure of bacterial cells	[102]
**Sulfur**	synthesis of proteins and chlorophyll; nitrogen use efficiency	[103]
	nodulation and BNF, biosynthesis intensity, and nitrogenase activity	[104]
	affects the content of leghemoglobin and glucose in nodules, ATP in mitochondria and bacteroids, and ferredoxin in bacteroids	[105,106,107,108,109]
**Calcium**	signaling between plants and bacteria and recognition of rhizobial nodulation factors	[44]
	increase in the level of flavonoids in plant root exudates	[110]
	increase release of bacterial exopolysaccharides	[42]
**Magnesium**	growth and development of roots and root nodules	[112]
	synthesis of protein	[112]
	metabolism and distribution of N; regulation C-N transport	[112,113,114]
**Iron**	leghemoglobin cofactor, cytochrome, and ferredoxin	[115]
	synthesis of chlorophyll, growth, and development of plants	[116]
**Molybdenum**	metabolic processes	[117]
	nitrogen reduction process	[118]
	growth and development of nodules; BNF	[119,120,122]
	affects the N content in plants	[121]
**Boron**	maintains the structure of Rhizobium cell membranes and for the exchange of signals between plants and bacteria	[123]
**Nickiel**	urease enzyme activator	[124]
	increasing the energy efficiency of BNF by utilization of gaseous hydrogen	[125]
**Cobalt**	growth and development of nodules; increase in the efficiency of symbiosis	[125]
	cobalamin component	[126]
	decrease in the concentration of reactive oxygen species	[127]
**Zinc**	catalytic cofactor and structural element in proteins	[128]
	deficiency limits the growth and development of plants	[129]
	reduction of nitrogenase activity	[130]
	increases the efficiency of metabolic processes and water use by plants	[131]
**Manganese**	participation in physiological and biochemical processes; conversion of nitrate (NO_3_^−^) to ammonia (NH_3_)	[125,132]
	limits the impact of reactive oxygen species on root nodules	[133]
	disrupts the formation of new root nodules and inhibits bacterial activity	[137,138,139,140,141]
**Copper**	enzyme cofactor, free radical metabolism	[125,136]
	disrupts the formation of new root nodules and inhibits bacterial activity and bactericidal effects	[137,138,139,140,141]

## Data Availability

No new data were created or analyzed in this study. Data sharing is not applicable to this article.
